# Robustness of the reproductive number estimates in vector-borne disease systems

**DOI:** 10.1371/journal.pntd.0006999

**Published:** 2018-12-17

**Authors:** Warren Tennant, Mario Recker

**Affiliations:** Centre for Mathematics and the Environment, University of Exeter, Penryn Campus, Penryn, United Kingdom; Imperial College London, Faculty of Medicine, School of Public Health, UNITED KINGDOM

## Abstract

**Background:**

The required efforts, feasibility and predicted success of an intervention strategy against an infectious disease are partially determined by its basic reproduction number, *R*_0_. In its simplest form *R*_0_ can be understood as the product of the infectious period, the number of infectious contacts and the per-contact transmission probability, which in the case of vector-transmitted diseases necessarily extend to the vector stages. As vectors do not usually recover from infection, they remain infectious for life, which places high significance on the vector’s life expectancy. Current methods for estimating the *R*_0_ for a vector-borne disease are mostly derived from compartmental modelling frameworks assuming constant vector mortality rates. We hypothesised that some of the assumptions underlying these models can lead to unrealistic high vector life expectancies with important repercussions for *R*_0_ estimates.

**Methodology and principal findings:**

Here we used a stochastic, individual-based model which allowed us to directly measure the number of secondary infections arising from one index case under different assumptions about vector mortality. Our results confirm that formulas based on age-independent mortality rates can overestimate *R*_0_ by nearly 100% compared to our own estimate derived from first principles. We further provide a correction factor that can be used with a standard *R*_0_ formula and adjusts for the discrepancies due to erroneous vector age distributions.

**Conclusion:**

Vector mortality rates play a crucial role for the success and general epidemiology of vector-transmitted diseases. Many modelling efforts intrinsically assume these to be age-independent, which, as clearly demonstrated here, can lead to severe over-estimation of the disease’s reproduction number. Our results thus re-emphasise the importance of obtaining field-relevant and species-dependent vector mortality rates, which in turn would facilitate more realistic intervention impact predictions.

## Author summary

Many infectious diseases of public health concern, such as dengue, Zika and malaria, are transmitted by insect vectors. Control effort required to curb their continued spread or even prevent their establishment in the first place are partially determined by the disease’s basic reproduction number, *R*_0_. Of particular importance for estimating *R*_0_ is the duration at which the vector can transmit the disease, which is limited by its life expectancy. Many *R*_0_ estimation methods are based on mathematical frameworks that assume constant vector mortality rates. Here we demonstrate how the resulting exponential distribution in life-expectancy can lead to significant over-estimations. By means of an individual-based model we elucidate the effect of vector mortality on *R*_0_ estimates and derive a correction factor that alleviates some of the discrepancies due to erroneous vector age distributions. Our results clearly demonstrate the need to obtain more realistic, i.e. field-relevant vector mortality rates in order to generate robust estimates of a disease’s reproduction number and provide guidance for setting-specific disease control.

## Introduction

Over the last few decades there has been a global rise in the emergence and re-emergence of vector-borne infectious diseases [1]. The continuing threat of *Plasmodium falciparum* malaria [2, 3] and dengue [4], the rapid, near pandemic spread of Zika virus [5] or the recent epizootic outbreak of *Yersinia pestis* (plague) in Madagascar [6] are just some examples of pathogens transmitted by insect vectors that pose a major threat to global public health. Their dependence on insects for transmission between vertebrate hosts has a number of important implications. First, they are frequently subject to strong spatial and temporal fluctuations due to environmental and climatic variations, such as seasonality in rainfall or temperature. Second, these pathogens should be amenable to vector control. That is, disease transmission can, at least in theory, be interrupted simply by removing the insect vector (e.g. use of insecticides) or by preventing contact between the vector and the host (e.g. use of bednets). Furthermore, it has been suggested that only a fraction of insects need to be removed or vector-host contacts to be prevented for the disease to die out. This concept is largely based on mathematical theory that can be traced back to the first formal description and mathematical treatment of the malaria life-cycle by Ross [7]. Unfortunately, translating theoretical predictions to practical applications, especially with regards to disease elimination through vector control, has only resulted in partial success.

The epidemiological reasoning behind the theory relies on a particular threshold condition involving the so-called basic reproduction number, *R*_0_, which denotes the expected number of secondary cases arising from a single infection in a totally susceptible population [8]. To date, *R*_0_ is frequently used either to predict the extent of an epidemic outbreak or to derive the necessary conditions to prevent this outbreak from happening, e.g. by means of vaccination. The crux of the problem is how to robustly derive or estimate this number in the first place. Compartmentalised systems of ordinary differential equations (ODEs) have been in use for decades to understand infectious diseases at the population level and provide the backbone for most formulas for *R*_0_ [9]. These allow the reproduction number to be computed either exclusively using empirically informed parameter estimates or from the initial growth rate of an outbreak [10]. Although the latter is the more common approximation method for directly-transmitted disease [11–14], it has equally been applied to vector-borne pathogens [15–17].

An important consideration for *R*_0_ estimates of vector-borne pathogens is that these can vary substantially across space and time. For example, reported *R*_0_ estimates for the complete transmission cycle of *Plasmodium falciparum* in Africa range from 1 to more than 3,000 [18, 19]. Based on nine epidemics in Brazil between 1996 and 2003, the reproduction number for dengue has been estimated to be somewhere between 2 to 103 [20], and median estimates for Zika range between 2.6–4.8 in French Polynesia [21] and 4–9 in Rio de Janiero [22]. The reasons for such wide variations are manifold. As mentioned earlier, the dependence on insect vectors for transmission can naturally introduce large spatio-temporal heterogeneities. That is, a disease introduced during the dry season will behave very differently to the same disease being introduced during the rainy seasons. Equally, an outbreak in a densely populated urban area will likely take a different course than an outbreak in a sparsely populated rural area. Here we argue that in addition to these natural variations and potential differences in data collection and analyses, the actual methodologies used to derive *R*_0_ estimates can also introduce substantial discrepancies.

A crucial component of the reproduction number for a vector-borne disease is the mean time that an infected vector is able to transmit to a host, or the infectious vector-to-host transmission period (VHTP) [23]. As infectious vectors are assumed to continue to transmit the disease until death, the VHTP is determined both by the life expectancy of the vector and the extrinsic incubation period of the pathogen. For mathematical simplicity, most epidemiological models of vector-borne diseases assume that vectors have a constant (daily) mortality rate. However, this assumption is in stark contrast to findings from lab-based and field mark-and-recapture studies. For example, survival probabilities of the dengue mosquito vector *Aedes aegypti* and the principal malaria vectors *Anopheles stephensi* and *An*. *gambia* have been shown to be strongly age-dependent [24–27]. Although it should be clear that current lab and field-based studies of vector survivorship come with their own set of limitations and uncertainties, constant, i.e. age-independent mortality rates are biologically less likely than assuming a general decrease in the survival probability with age.

Previous work has looked into the effects of logistic mortality rates on the vectorial capacity, the mosquito-related components of *R*_0_ [28]. However, the effects of assuming constant vector mortality on *R*_0_ in a system where death rates are strongly age-dependent have not yet been explored. Here we compared a commonly used *R*_0_ formula based on continuous-time differential equation model using constant mortality rates to an *R*_0_ estimate derived from first principles under relaxed assumptions about vector mortality. Using a stochastic, individual-based simulation model (IBM), which permits the direct measurement of the average number of secondary cases, we demonstrate how the underlying assumptions of vector survivorship can significantly inflate *R*_0_ estimates. We further show how estimates based on endemic equilibria are generally more robust and derive a correction factor to ameliorate *R*_0_-inflations in estimation methods based on epidemic growth curves.

## Methods

### Model frameworks

We derived *R*_0_ estimates from two different epidemiological frameworks: (i) a simple, single-strain vector-borne disease model based on ordinary differential equations (ODE), where vector mortality is assumed to be constant, leading to an exponentially distributed vector survivorship, and (ii) a stochastic individual-based model (IBM), which permits more explicit control over the demographic processes regulating birth and death rates.

#### ODE model

The classical ODE approach to model infectious diseases is obtained by dividing the population into those that are susceptible (*S*), exposed but not yet infectious (*E*), infectious (*I*) and recovered (*R*). The same principle is then applied to extend these models to vector-transmitted diseases, except for the fact that vectors usually do not recover from infection but are instead assumed to remain infectious until death. This model can be realised by the following set of differential equations

$$\begin{array}{c} \frac{dS_{H}}{dt}=\mu_{H}N_{H}-p_{H}\beta I_{V}\frac{S_{H}}{N_{H}}-\mu_{H}S_{H} \end{array}$$ (1)

$$\begin{array}{c} \frac{dE_{H}}{dt}=p_{H}\beta I_{V}\frac{S_{H}}{N_{H}}-\epsilon_{H}E_{H}-\mu_{H}E_{H} \end{array}$$ (2)

$$\begin{array}{c} \frac{dI_{H}}{dt}=\epsilon_{H}E_{H}-\gamma I_{H}-\mu_{H}I_{H} \end{array}$$ (3)

$$\begin{array}{c} \frac{dR_{H}}{dt}=\gamma I_{H}-\mu_{H}R_{H} \end{array}$$ (4)

$$\begin{array}{c} \frac{dS_{V}}{dt}=\mu_{V}N_{V}-p_{V}\beta S_{V}\frac{I_{H}}{N_{H}}-\mu_{V}S_{V} \end{array}$$ (5)

$$\begin{array}{c} \frac{dE_{V}}{dt}=p_{V}\beta S_{V}\frac{I_{H}}{N_{H}}-\epsilon_{V}E_{V}-\mu_{V}E_{V} \end{array}$$ (6)

$$\begin{array}{c} \frac{dI_{V}}{dt}=\epsilon_{V}E_{V}-\mu_{V}I_{V} \end{array}$$ (7)

**Fig 1:**
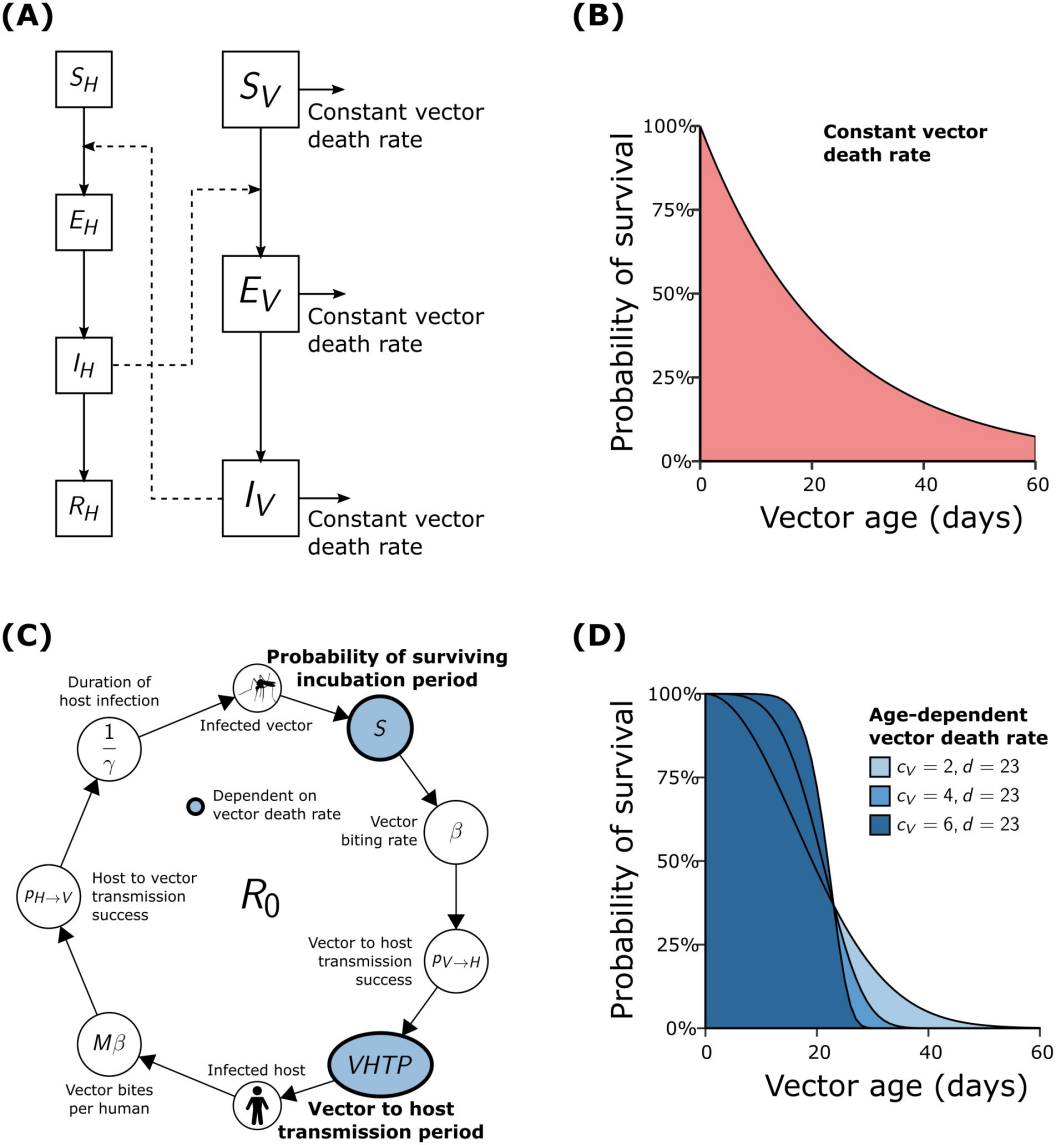
Comparison of flow diagrams and vector death rates between the ODE and IBM frameworks. **(A)** The compartmentalised system of differential equations for a vector-borne pathogen assumes constant vector mortality rates from each state of infection. **(B)** Constant vector mortality rates result in exponential age distribution of vectors (with 1/*μ*_*V*_ = 21.3), with a high proportion of individuals living far beyond their life-expectancy. **(C)** The transmission cycle of a vector-borne pathogen used in the individual-based model highlighting the dependency of the infectious period and the probability of surviving the extrinsic incubation period on the mortality rate of vectors. **(D)** The age distribution of vectors under three different Weibull distributed mortality risks with an increasing dependency on vector age (light blue to dark blue with *c*_*V*_ = 2, 4, 6 and *d*_*V*_ = 23).

Here, 1/*μ*_*H*,*V*_ are the mean host and vector life expectancies; *β* is the daily biting rate; *p*_*H*,*V*_ are the per-bite transmission probabilities from vector to human and human to vector, respectively; 1/*ϵ*_*H*,*V*_ are the incubation periods in the host and vector, respectively; and 1/*γ* is the mean infectious period in the host. This model is illustrated by means of a flow diagram in Fig 1A.

#### Stochastic IBM

We employed a spatially-explicit IBM (motivated by the one previously proposed by [29]), which, similarly to the ODE approach, assumes that individual hosts are either susceptible (*S*_*H*_), exposed (*E*_*H*_), infectious (*I*_*H*_), or recovered (*R*_*H*_). Individual vectors are equally set to be either susceptible (*S*_*V*_), exposed (*E*_*V*_) or infectious (*I*_*V*_), in which state they remain until death. Vectors are assumed to bite at a constant per day rate *β*. Infectious vectors transmit the infection to a host with probability *p*_*H*_, and susceptible vectors become infected upon biting an infectious host with probability *p*_*V*_. For simplicity we fixed the extrinsic incubation period (1/*ϵ*_*V*_ days), the intrinsic incubation period (1/*ϵ*_*H*_ days) as well as the length of infections in the host (1/*γ* days).

In contrast to the ODE model, we assumed age-dependent mortalities for both the hosts and the vectors, governed a Weibull distribution:

$$\begin{array}{c} \mu_{i}(t)=\frac{c_{i}}{d_{i}^{c_{i}}}t^{c_{i}-1}e^{-(\frac{t}{d_{i}}{)}^{c_{i}}} \end{array}$$ (8)

where *c*_*i*_ and *d*_*i*_ are the scale and shape parameters, with *i* ∈ {*H*, *V*} denoting the parameters for host and vectors, respectively. Setting *c*_*V*_ = 1 results in an exponential vector age distribution (Fig 1B), i.e. where a vector’s risk of death is age-independent equivalent to the above ODE formulation. Defining *c*_*V*_ > 1 results in a sigmoid age profile (Fig 1D) with the vector death rate being age-dependent. This allowed us to investigate the effect of different vector age distributions on the reproduction number *R*_0_ within this framework.

The host and vector populations were divided into a set of communities organized into a lattice. The distribution of humans and vectors was uniform across all communities, and it was assumed that individuals mix homogeneously within each community. Human movement was incorporated by allowing human individuals to temporarily visit any sub-population in the lattice with probability *ω*. The remaining proportion of transmission events from each community, 1 − *ω*, were dispersed to surrounding local sub-populations, thereby modelling the movement of vectors. Please refer to S1 Text for a more detailed description of the individual-based model.

### Estimation of *R*_0_

#### ODE-based reproduction number

We derived the *R*_0_ estimates from the ODE model by applying the next generation approach [30], which relates the number of newly infected individuals in the compartments in consecutive generations to one another (see S1 Text for details), yielding

$$\begin{array}{c} R_{0}=\frac{Mp_{H}p_{V}\beta^{2}}{\left(\gamma +\mu_{H}\right)}\frac{1}{\mu_{V}}\frac{\epsilon_{H}}{\epsilon_{H}+\mu_{H}}\frac{\epsilon_{V}}{\epsilon_{V}+\mu_{V}} \end{array}$$ (9)

where *M* is the vector:host ratio (*N*_*V*_: *N*_*H*_) and $\frac{\epsilon_{H}}{\epsilon_{H}+\mu_{H}}$ and $\frac{\epsilon_{V}}{\epsilon_{V}+\mu_{V}}$ are the probabilities of hosts and vectors surviving the intrinsic and extrinsic incubation period of the pathogen respectively.

For many vector-borne disease systems, such as malaria and dengue, both the human recovery rate, 1/*γ*, and the intrinsic incubation period 1/*ϵ*_*H*_, are much shorter than the mean human life expectancy, 1/*μ*_*H*_. We can therefore make the following approximation of the above formula, which, here, we define as $R_{0}^{\text{ODE}}$.

$$\begin{array}{c} R_{0}^{\text{ODE}}=\frac{Mp_{H}p_{V}\beta^{2}}{\gamma }\frac{1}{\mu_{V}}\frac{\epsilon_{V}}{\epsilon_{V}+\mu_{V}} \end{array}$$ (10)

#### IBM-based reproduction number

The basic reproduction number for the individual-based model, $R_{0}^{\text{IBM}}$, was derived from first principles using the transmission cycle of the pathogen, similar to [18] (Fig 1C). Starting with an infected host in an entirely susceptible population, this individual will infect on average *Mp*_*V*_ *β* vectors per day and will remain infected for 1/*γ* days. Therefore, a single infected host is expected to infect a total of *Mp*_*V*_ *β*/*γ* vectors.

A single infected vector will infect on average *p*_*H*_*β* hosts per day (in a totally susceptible population). As vectors remain infectious for the rest of their lives, the infectious period is defined as the difference between the mean life expectancy, $1/\mu_{I_{V}}$, and the mean age at which a vector becomes infectious, $1/\alpha_{I_{V}}$ (see S1 Text for details), meaning that an infectious vector will have $\left(1/\mu_{I_{V}}-1/\alpha_{I_{V}}\right)$ days to infect hosts.

Furthermore, the proportion of infected vectors that survive the extrinsic incubation period, denoted by $\rho_{E_{V}\to I_{V}}$, also depends on the vector mortality risk (see S1 Text for details). Combining all these terms, the basic reproduction number of the individual based model can be derived as

$$\begin{array}{c} R_{0}^{\text{IBM}}=\frac{Mp_{H}p_{V}\beta^{2}}{\gamma }(\frac{1}{\mu_{I_{V}}}-\frac{1}{\alpha_{I_{V}}})\rho_{E_{V}\to I_{V}} \end{array}$$ (11)

Note, the first term is identical to the first term of $R_{0}^{\text{ODE}}$. However, the second term, which denotes the infectious period of the vector, and the third term, which denotes the probability of vectors surviving the incubation period, differ between $R_{0}^{\text{ODE}}$ and $R_{0}^{\text{IBM}}$. This is because the formula for the reproduction number derived from the transmission cycle in the individual based model takes into account alternative (Weibull distributed) vector mortality risks, whereas the ODE system assumes a constant mortality rate (an exponential distributed mortality risk).

#### Timeseries-based reproduction number

In addition to the direct *R*_0_ formulas derived above, we also considered two common methods to estimate the reproduction number from timeseries data: one approach based on the initial growth rate of an epidemic outbreak (S1A Fig), and one based on the dynamic equilibrium of an endemic scenario (S1B Fig). Both epidemic and endemic cases were simulated using the individual based model.

*Epidemic growth rate*. The epidemic outbreak method for estimating the reproduction number requires timeseries data for the introduction of the disease into a completely susceptible population [31]. The initial (exponential) growth rate, λ, was obtained by fitting a Poisson generalised linear model to the initial outbreak data (S1A Fig). Using the classical SEIR-SEI system of ordinary differential equations for a vector-borne disease, the formula for the basic reproduction number, $R_{0}^{\lambda }$, can be derived as

$$\begin{array}{c} R_{0}^{\lambda }=(1+\frac{\lambda }{\gamma })(1+\frac{\lambda }{\mu_{V}})\exp(\lambda (\frac{1}{\epsilon_{V}}+\frac{1}{\epsilon_{H}})) \end{array}$$ (12)

where 1/*μ*_*V*_ is the mean life expectancy of the vector (see S1 Text for derivation).

*Endemic equilibrium approach*. The asymptotically stable steady state of susceptible individuals in an ODE-based SEIR system for a directly transmitted disease can be used to estimate the basic reproduction number [10] as

$$\begin{array}{c} R_{0}^{*}=\frac{N_{H}}{S^{*}} \end{array}$$ (13)

where S* is the number of susceptible individuals at equilibrium. The directly transmitted disease *R*_0_ estimate was then used as an approximation for the basic reproduction number of an endemic vector-borne disease. For the stochastic IBM this required the system to reach a dynamic equilibrium, where the proportion of susceptibles oscillates around the deterministic equilibrium (as the inherent stochasticities prevent the system from reaching an equilibrium state). We calculated $R_{0}^{*}$ using susceptibility levels at a single time point, as well as the mean proportion of susceptible individuals over the final five years of the timeseries (S1B Fig).

### Parameter values

Table 1 provides an overview of the parameters and parameter values used throughout this work (unless stated otherwise). The values were chosen to reflect the epidemiological dynamics of an arboviral disease, such as dengue or Zika. However, the results presented here are qualitatively independent of the particular choice of parameters; S2 Fig show the results of model sensitivity analyses with respect to the dependency of *R*_0_ estimates on particular parameter values.

**Table 1:** The default set of parameter values used in the simulation of the individual-based model describing the spread of a vector-borne disease.

Parameter	Description	Value [range^†^]
|*C*|	Number of communities in lattice	400 [1, 16384]
*N*_*H*_	Host population size	100000
*M*	Vector to host ratio	1.2
*c*_*H*_	Host mortality shape parameter	6
*d*_*H*_	Host mortality scale parameter	75 × 365 days
*c*_*V*_	Vector mortality shape parameter	4 [1–4]
*d*_*V*_	Vector mortality scale parameter	23 days [10, 40]
1/*μ*_*H*_	Mean human life expectancy in ODE	70 years
1/*μ*_*V*_	Mean vector life expectancy in ODE	21.3 days
1/*γ*	Host recovery time	4 days
1/*ϵ*_*H*_	Intrinsic incubation period	6 days
1/*ϵ*_*V*_	Extrinsic incubation period	5 days
*β*	Per day contact rate	0.6 days^−1^
*p*_*H*_	Pathogen transmission success to host	0.5
*p*_*V*_	Pathogen transmission success to vector	0.5
*σ*	Local disease dispersal kernel standard deviation	2
*ω*	Long distance transmission probability	10^−4^
*ι*	External infection rate per 100,000 hosts per day	10^−2^ [10^−5^, 1]

^†^ range considered for sensitivity analyses.

## Results

A multitude of the mathematical models put forward to study the dynamics of vector-borne diseases are based on compartmental models described by systems of ordinary differential equations (ODE). Crucial to these types of models is the assumption of constant death rates. As vectors are assumed to remain infectious for life, such assumptions influence not only the resulting dynamics but also the estimates of the disease’s basic reproduction number *R*_0_ (and relatedly the (time-varying) effective reproduction number *R*_*e*_(*t*)). Here we aimed to quantify the effects of relaxing the assumption of constant vector death rate on *R*_0_ estimates within the same theoretical setting. This was done by comparing the *R*_0_ values derived from an SEIR-SEI system of ODEs with a formula derived from first principles using the transmission cycle of a generic vector-borne disease using different assumptions about vector mortality rates (see Methods). We then verified these estimates by means of a stochastic individual-based model, which allowed us to directly *measure* *R*_0_ from running repeat simulations of introducing an infected individuals into a fully susceptible population. The same model was also used to derive *R*_0_ estimates from simulated timeseries data.

### Constant vector mortality rates over-estimate *R*_0_

Assuming constant vector mortality rates leads to exponentially distributed age profiles (Fig 1B), which permit some vectors to live in excess of four times their mean life expectancy and potentially to transmit the pathogen for an unusually long period of time. Even more concerning is that the vector life expectancy in each compartment of the infection process is essentially the same (Fig 1A). That is, independent of when in its life a vector becomes infected and infectious, its remaining life expectancy remains exponentially distributed around the mean life expectancy. As a consequence, all infectious vectors have a vector-to-human transmission period (VHTP) equal to the mean life expectancy of all vectors, 1/*μ*_*V*_, with obvious consequences for *R*_0_ estimates.

In contrast to ODE models, individual-based models (IBM) permit much greater control over vector mortality rates. Here we used (Weibull distributed) age-dependent vector death rates (see Methods), which yield a range of sigmoid age profiles (Fig 1D) but which all prevent vectors from living severely extended lives. More importantly, an individual vector’s remaining life expectancy remains unchanged when transitioning between susceptible and infected state or between infected and infectious state, resulting in shorter and more realistic infectious periods.

We demonstrate the effect of assuming different vector mortality rates by comparing the *R*_0_ estimates derived from the ODE model to the individual-based model (see Methods). As expected, using parameters as listed in Table 1 we find that the reproduction numbers from the ODE and IBM systems are similar under the assumption of constant vector mortality rates (Table 2). The small discrepancy between the two models is due to the ODE model’s assumption of an exponentially distributed extrinsic incubation period, whereas the IBM assumes this to be a fixed length of time. Using the IBM approach to track individual mosquitoes and infection events we also find that under this assumption the mean age at which vectors become infected is 20 days and infectious at an age of 25 days, i.e. days beyond their average life-expectancy. Furthermore, those vectors that have become infectious live for an average of 46 days, which means that their infectious period is 21 days (equal to the life expectancy of all vectors). This clearly highlights the discrepancy between model outputs based on constant mortality rates and biological reality. In contrast, assuming age-dependent mortality rates (Weibull shape parameter, *c*_*V*_ = 4) results in biologically more reasonable infectious periods of 11 days (Table 2) and *R*_0_ estimates that are less than half of those based on a model with constant mortality.

**Table 2:** Comparison of the reproduction numbers derived from a system of ordinary differential equations and the individual-based model under the assumption of constant versus age-dependent mortality rates.

Model	Vector death rate	Life expectancy	Infectious period	*R*_0_
ODE	Constant	21 days	21 days	7.5
IBM	Constant	21 days	21 days	7.2
IBM	Age-dependent	21 days	11 days	3.2

**Fig 2:**
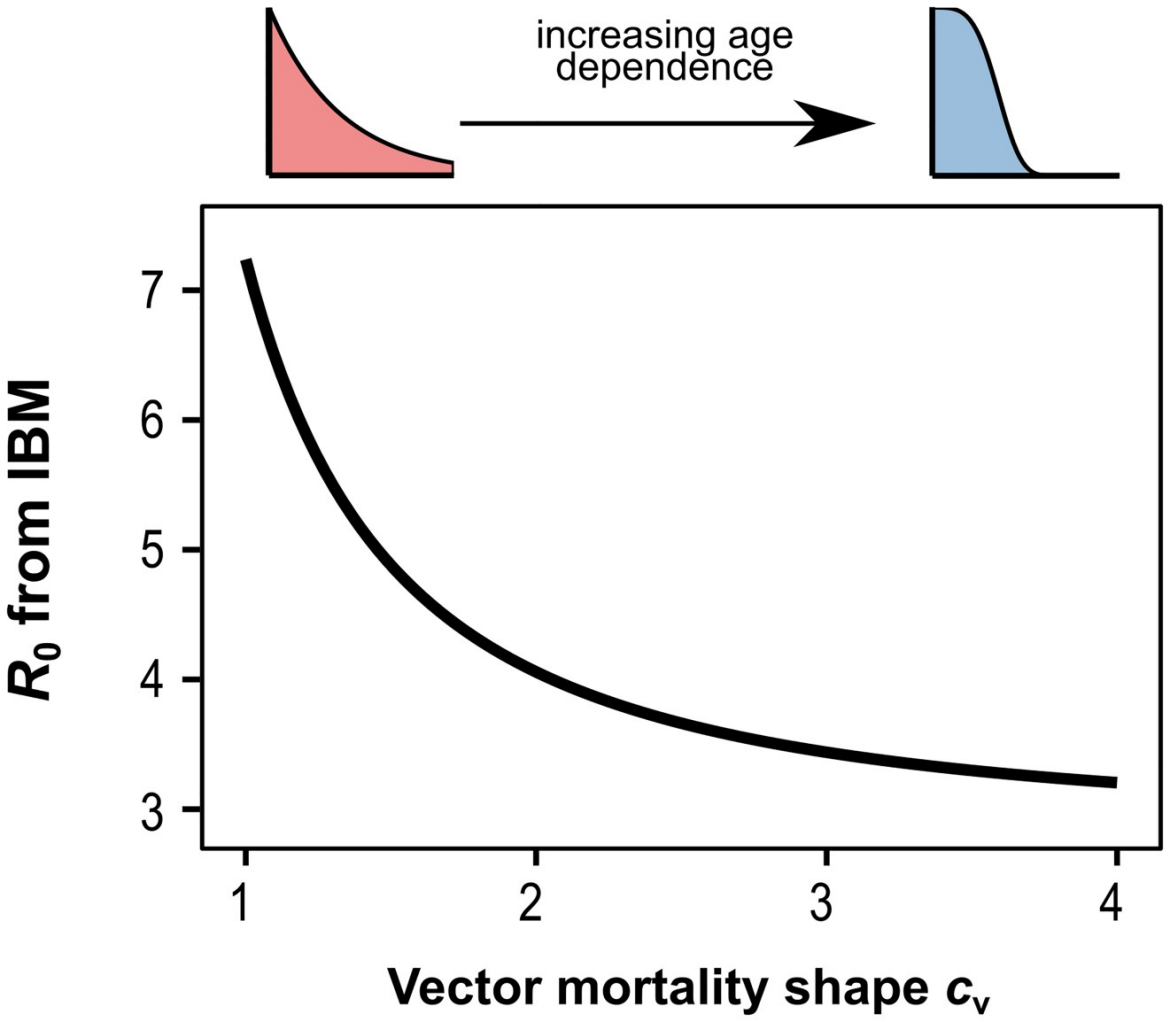
The effects of age-dependent mortality rates on the reproduction number. Starting by assuming a constant death rate (*c*_*V*_ = 1), the reproduction number derived from the transmission cycle of the individual based model, $R_{0}^{\text{IBM}}$, rapidly decreases as vector mortality becomes increasingly more age-dependent (*c*_*V*_ > 1) under constant average life spans.

To further demonstrate the dependency of *R*_0_ on different distributions of mosquito survivorship, we changed the Weibull distribution of vector mortality to transition smoothly between an exponential (*c*_*V*_ = 1) and a sigmoid (*c*_*V*_ > 1) age profiles and by keeping the average life expectancy constant. As illustrated in Fig 2, relaxing the assumption of constant mortality and resultant exponential age profile shortens the average infectious period and lowers the reproduction number as derived from the transmission cycle of the pathogen, i.e. $R_{0}^{\text{IBM}}$. This clearly demonstrate that as well as the vector life expectancy, the actual shape of the survival curve strongly determines the estimated values of a pathogen’s reproduction number.

### Comparing *R*_0_ estimates through direct measurement

The scenario defined by the reproduction number, whereby a single infectious case enters an entirely susceptible population, is arguably unrealistic for most diseases. Furthermore, disease transmission is an inherently stochastic process, such that each realisation of a disease introduction event is likely to take a different course. We should therefore expect that *R*_0_ estimates derived from such introductory events should come with a certain degree of variation. In order to better understand the variability of the expected number of secondary cases and then to directly compare the above formula-based *R*_0_ estimates, we simulated disease introduction events into a completely susceptible population using our IBM framework and kept track of all secondary host infections resulting from the index case.

**Fig 3:**
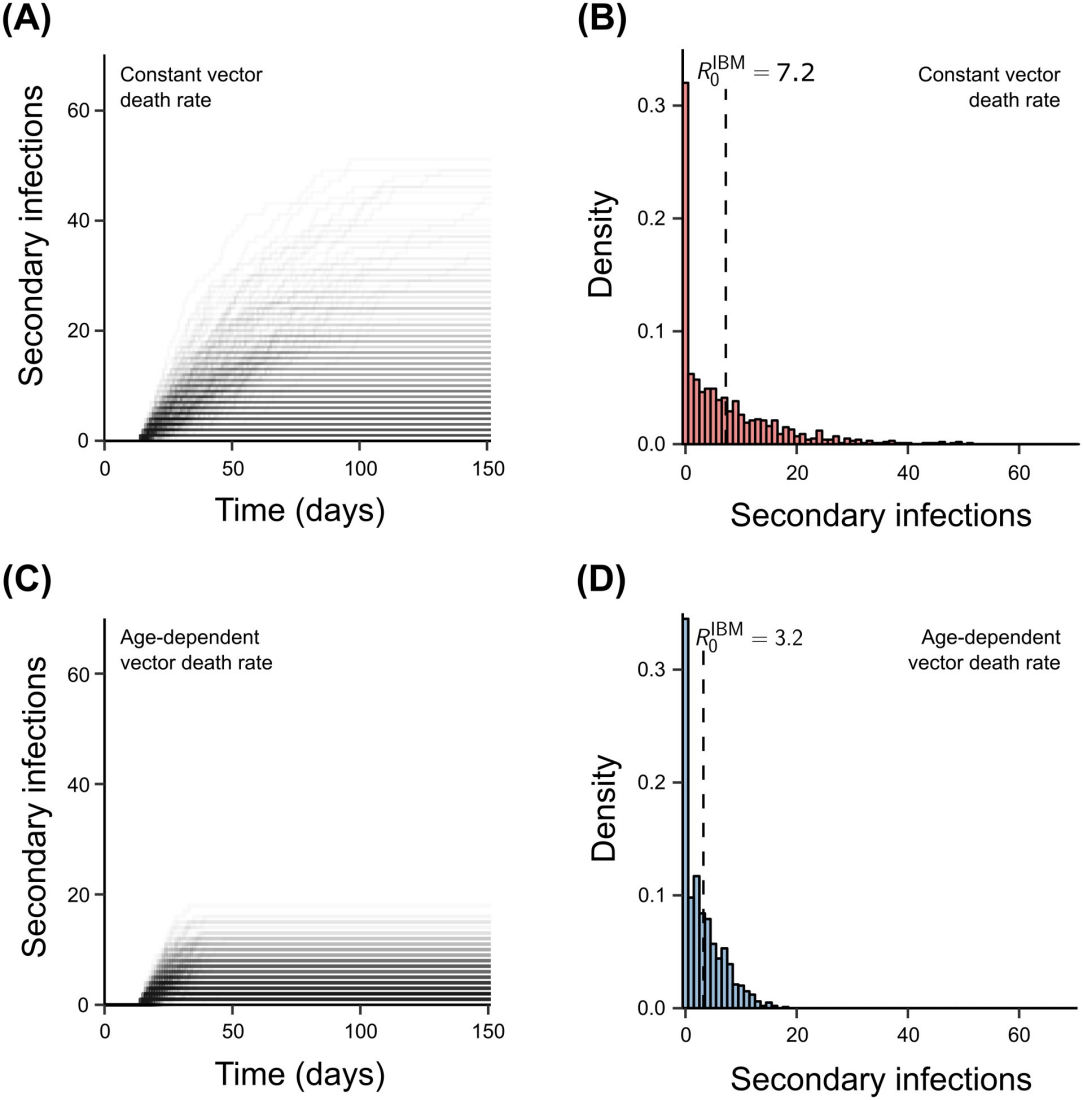
Reproductive number measured from the individual-based model. **(A,C)** Keeping track of the number of secondary infections in an entirely susceptible population starting with a single human case over time illustrates how in the model with constant vector death rates, secondary cases can still occur more than 100 days after the disease is introduced. This is in stark contrast to the model with age-dependent mortality, where most secondary infections occur within the average life-expectancy of the mosquito. Each solid line represents the accumulation pathway of secondary infections over time with darkness indicating the percentage of simulations that follow each pathway. The individual-based model was executed 500 times yielding a distribution of total number of secondary infections, or *R*_0_, assuming either constant **(B)** or age-dependent vector mortality rates **(D)**. The two dotted lines are the reproduction numbers calculated from the theoretical IBM calculation. The mean number of secondary infections was comparable to the reproduction number derived from the transmission cycle of a vector-borne pathogen. Results are based on 500 model runs. Parameter values as in Table 1, except *c*_*v*_ = 1, *d*_*v*_ = 20.8 (constant mortality) and *c*_*v*_ = 4, *d*_*v*_ = 23 (age-dependent mortality).

As before we compared the two different assumptions regarding vector life expectancy: constant vs. age-dependent mortality rates. As shown in Fig 3, there is a wide distribution in the number of secondary infections, particularly when we assumed constant vector death rates (Fig 3A and 3B). In that case it was not unusual to observe 40-60 secondary infections, due to the aforementioned unrealistically high life-expectancies for some of the vectors, permitting the accumulation of secondary cases well after the primary human case has recovered (Fig 3A). The mean number of secondary infection (i.e. *R*_0_) across 500 model simulations was around 7, more than twice that of the model which assumed age-dependent mortalities. In the latter case we observed secondary infections in the range of 0 to 18 (due to the model’s stochastic nature where some vectors may be infected for their entire life) and with a mean of around 3.2 (Fig 3C and 3D), in line with theoretical expectations. Please refer to S3 Fig for sensitivity on model parameters on the direct measurement of mean secondary infections from the IBM.

An interesting observation is that under both assumptions of vector mortality, over 30% of our simulations resulted in zero secondary infections, as either the single primary case did not infect any vectors, the infected vectors failed to survive the extrinsic incubation period, or the infectious vectors failed to transmit the pathogen. Shorter infectious periods for both the host and the vector, a longer extrinsic incubation period, and lower transmissibility naturally decrease the overall likelihood of transmission from primary to secondary cases. Therefore, the proportion of failed outbreaks crucially depends on all these factors (S4 Fig).

### Initial growth rate methods can lead to over-estimation of *R*_0_

In most cases, only successful disease introductions that lead to epidemic outbreaks are observed. These outbreaks can then be used to estimate the reproduction number based on the initial epidemic growth rate λ (see Methods). Formulas to calculate *R*_0_ from λ are usually based on ODE modelling frameworks assuming constant vector death rates. To investigate the effect of this assumption on estimating a disease’s basic reproduction number from epidemic growth rates, $R_{0}^{\lambda }$, we used our IBM framework to generate 100 epidemic outbreaks (discounting failed introductory events) under identical initial conditions for both constant and age-dependent mortality rates.

**Fig 4:**
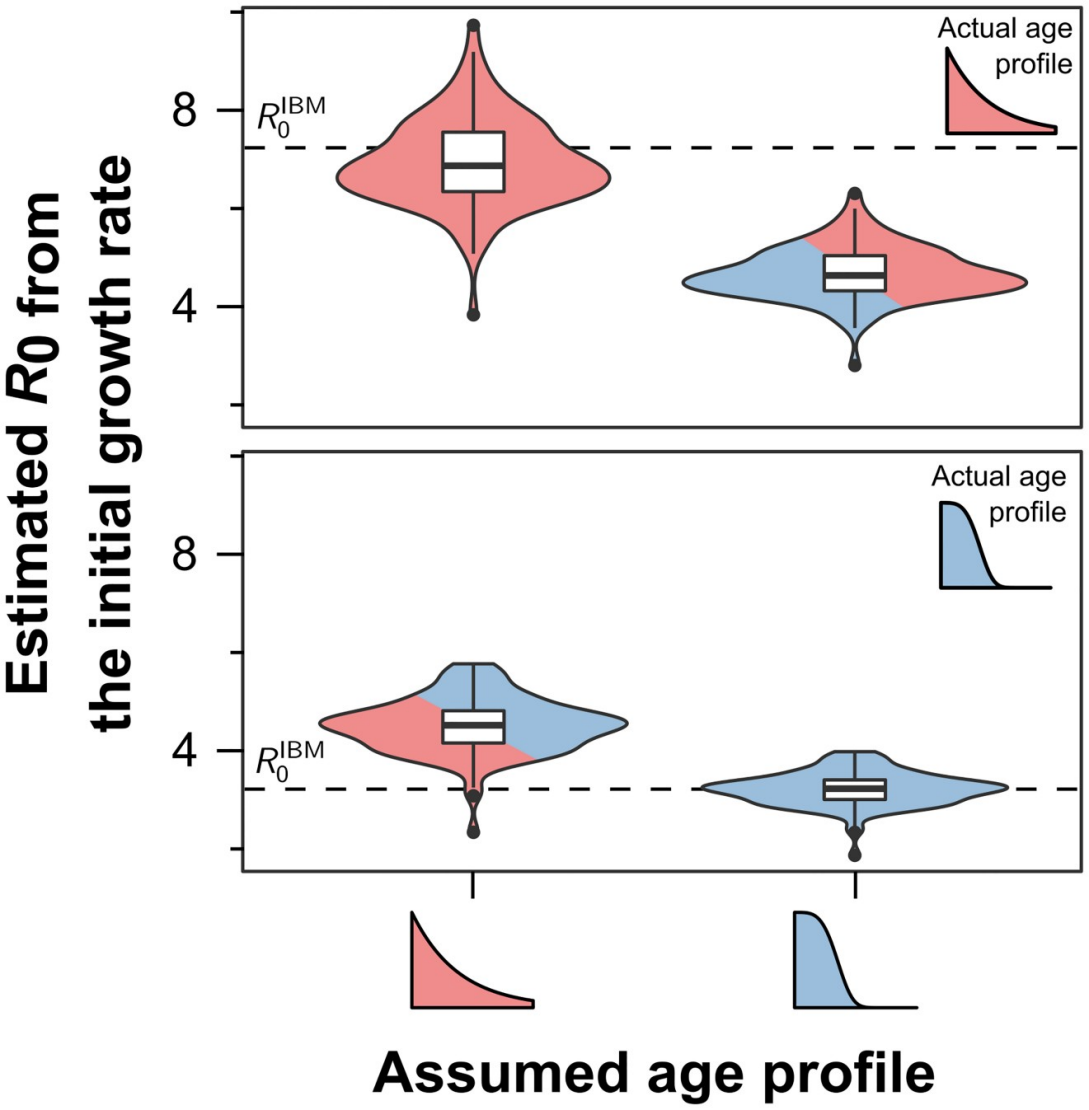
*R*_0_ estimates based on the initial growth can over-estimate *R*_0_. Violin plots showing the density distribution of *R*_0_ estimates based on the initial epidemic growth rates. When the model assumptions regarding vector mortality correctly reflect the true mortality rates (solid colour) we find that the method based on epidemic growth generates fairly robust estimates of the true *R*_0_. When the assumed mortality rate does not coincide with the real one (mixed colour), estimates can be off by a wide margin. Results are based on 100 model runs for each scenario; the inserted boxplots indicate the median and interquartile ranges.

As illustrated in Fig 4A, estimating the reproduction number from initial outbreak data is fairly reliable as long as the empirical age profiles of the mosquitoes match the one assumed in the model. That is, if mosquito mortality was indeed independent of age, leading to exponentially distributed age profiles, then $R_{0}^{\lambda }$ can provide good estimates of the real reproduction number. However, if the risk of dying does increase with age, then $R_{0}^{\lambda }$, as derived form the ODE framework, is once again significantly over-inflated. Likewise, assuming age-dependent death rates when mortality is in fact constant, this could lead to an underestimation of the true reproduction number; note, however, that the latter scenario is arguably less relevant in biological terms.

Noticeable in all situations is the considerable variance in $R_{0}^{\lambda }$. This is due to the stochastic nature of our spatial IBM framework, which to a certain extent should also reflect the natural stochasticities underlying real vector-borne disease systems. Changing the model’s spatial and demographic set-up will obviously affect the variance reported here; however, the results, related to the mean values, are to be understood as independent of the model’s underlying structure.

### Correction for initial growth rate methods

As shown in Fig 4, using the initial epidemic growth rate is only appropriate when empirical vector mortality is indeed age-independent, whereas it can lead to significant over-estimations otherwise. In order to compensate for this and include age-dependent vector mortality rates into the ODE-derived formula for $R_{0}^{\lambda }$, we replaced this critical term by the vector to host transmission period (VHTP), denoted by $\nu_{I_{V}}$, calculated directly from an assumed vector age profile (see Methods), which yields the corrected estimate

$$\begin{array}{c} {\hat{R}}_{0}^{\lambda }=(1+\lambda \nu_{I_{V}})(\frac{\mu_{V}}{\mu_{V}+\lambda })R_{0}^{\lambda } \end{array}$$ (14)

where *μ*_*V*_ is the constant vector mortality rate in the classical system of ordinary differential equations. Crucially, a vector age profile has to be assumed explicitly to calculate the VHTP. And as before, if the assumed profile in ${\hat{R}}_{0}^{\lambda }$ matches the simulations’ profile, we find that the derived reproduction numbers are good estimates of the actual ones, with the same variance as before (Fig 4).

### Endemic equilibrium can provide robust estimates of *R*_0_

**Fig 5:**
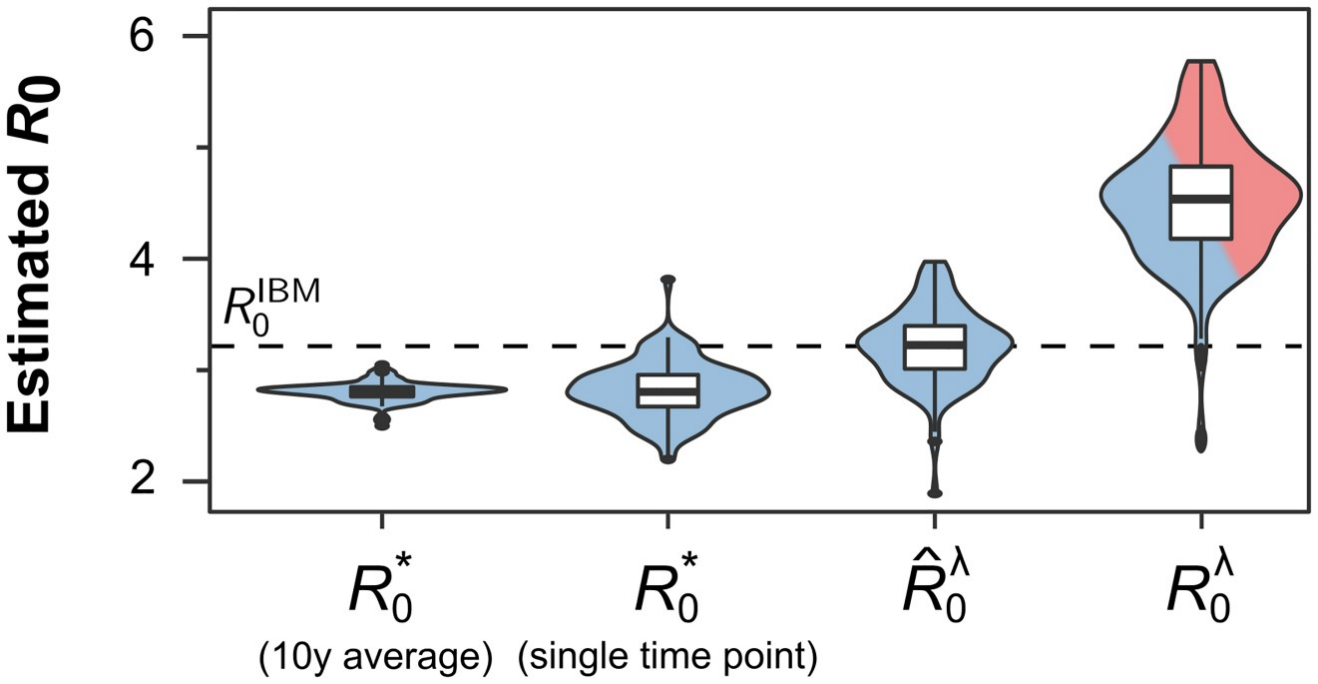
*R*_0_ estimates from the endemic equilibrium are more robust. Estimating *R*_0_ from the endemic equilibrium distribution of susceptibles ($R_{0}^{*}$) requires no assumptions about underlying vector survival rates and proves more robust than estimates based on the initial growth rate ($R_{0}^{\lambda }$), especially when using longitudinal data (compare single time point with 10 year average). Note that corrected values of $R_{0}^{\lambda }$, ${\hat{R}}_{0}^{\lambda }$, can yield good estimates but are still subject to significant variations around the mean. Results are based on 100 model runs for each scenario; the inserted boxplots indicate the median and interquartile ranges, and the dashed line denotes the theoretical *R*_0_ value.

Finally, we sought to estimate *R*_0_ from the dynamic equilibrium distribution of susceptibles in the human population (see Methods). Crucially, this approach does not require any *a priori* knowledge of mosquito survivorship and should therefore provide more robust estimates regardless of the underlying assumptions regarding vector mortality rates. Indeed, and as demonstrated in Fig 5, using the endemic state can provide reasonable estimates of a disease’s true (i.e. theoretical) *R*_0_ value, even though the formula itself was derived from a directly transmittable disease, which might explain why $R_{0}^{*}$ slightly underestimates *R*_0_.

As before we find a significant degree of variation around the mean estimates, due to the stochastic nature of disease transmission. This can somewhat be reduced by taken longer term averages (compare single time point estimates with 10 year average in Fig 5), which in reality will be limited due to data availability. Equally, the model and population structure itself, including as population size, importation rates, spatial structuring and mixing, all affect the stability of the dynamic equilibrium and with it the variance and hence robustness of $R_{0}^{*}$ (see S2 Fig). Although this method is only applicable for diseases that have reached at least a semi-endemic state, its parameter and assumption-free approach means that it should be considered as one of the most robust ways to estimate a disease’s reproduction number.

## Discussion

Mathematical models describing the population dynamics of an infectious disease provide the necessary frameworks by which we can calculate an infectious disease’s reproduction number, *R*_0_, based on specific parameters related to infection and transmission probabilities. One of the most important factors influencing *R*_0_ is the length at which an individual remains infectious. For vector-transmitted diseases this places huge significance on vector mortality rates as vectors usually do not clear an infection and instead remain infectious for life. Many formulas to estimate *R*_0_ are based on systems of ordinary differential equations (ODEs), which commonly assume that vector mortality is constant, i.e. independent of age. As we have demonstrated here, the resulting exponential distribution and the effective *resetting* of life expectancies as individuals transition through the infection stages permit some vectors to live for an extraordinary length of time. As a result, vectors are potentially able to transmit the disease multiple times that of what should biologically be possible, leading to significantly inflated *R*_0_ estimates.

In comparison to ODE models, individual-based models (IBMs) provide much greater control over the dynamics that govern both demography and disease transmission. Here we used an individual-based modelling approach to elucidate the influence of vector mortality on *R*_0_ estimates and to highlight the discrepancy between model predictions based on constant vs. age-dependent mortality. Because individual infection events can easily be tracked within an IBM, the basic reproduction number can essentially be measured simply by counting the number of secondary infections arising from a single index case. This in turn not only allowed us to compare different formulas for estimating *R*_0_ but also provided us with a better understanding of the degree of uncertainty surrounding these estimates.

As demonstrated here, the assumption of constant vector death rates can lead to significant over-estimation of *R*_0_. Importantly, it is not so much that the formulas commonly used to estimate *R*_0_ are inherently wrong but rather that the underlying assumption of the models from which they are derived are not necessarily aligned with biological reality. We found that one of the most robust methods to estimate a pathogen’s *R*_0_ is based on the proportion of susceptible individuals at endemic equilibrium, as this is entirely parameter free and does not require any assumption about vector death rates. Unfortunately, this only works for diseases that are well established in a population, and its reliability is strongly dependent on the stochasticity of the underlying endemic equilibrium, i.e. the (multi-annual) variations around the mean. For emerging diseases this is obviously not practical and estimation methods in those cases usually make use of epidemic growth curves instead. However, these also implicitly assume exponential vector age profiles and are therefore subject to inflation. In order to account for this we have here derived a correction factor that can be applied to classical *R*_0_ estimation formulas and which adjusts for most of the discrepancy between the vector-to-human transmission period (VHTP) of the biological system and the assumed system with constant vector mortality.

In this work we made use of an individual-based modelling framework to test the effect of non-exponential vector age distributions on *R*_0_. Alternative methods that allow for the (partial) relaxation of the assumption regarding constant mortality or vector senescence have also been proposed, including lumped-age class models [32] or systems of partial differential equations [33]. However, these methods can still suffer from the same issues as simpler ODE models, where transition rates between life and infection stages are usually exponentially distributed and where information about individual ages is lost at every transition stage. The ease at which different distributions that govern host and vector mortality, infection recovery and other epidemiological factors can be incorporated, make IBM frameworks the natural choice to examine the influence of vector mortality or other such factors on *R*_0_ estimations. Here we only concentrated on the effect of vector mortality, whereas similar arguments are equally valid for the distribution underlying the extrinsic incubation period [34], for example. Nevertheless, our work strongly suggests that vector mortality rates, or rather our assumptions about the age-dependency of survivorship, are the predominant factors, as our correction term for *R*_0_ estimates based on epidemic growth essentially recovers the true value.

Another important observation from this study was that when simulating the spread of a disease from a single infected individual and then calculating *R*_0_ based on the number of secondary infections, the stochastic nature of such events resulted in very wide distributions in *R*_0_. Although the assumption of age-dependent mortality rates generally prevented extremely high values of secondary cases, and therefore *R*_0_, the variance was still in the region of twice the mean and included a significant proportion of zero cases. That is, in around a third of the simulations we observed no secondary case at all despite starting off with the same initial conditions. This then begs the question whether these events should be counted towards the estimated *R*_0_ or not, as in reality we never observe such failed introductions. Comparing the expected with the observed *R*_0_ value would suggest that zero cases should be counted, which on the other hand implies that even high values of the reproduction number are by no means a guarantee that an outbreak should ensue if a disease gets introduced in a fully susceptible population (sufficient conditions to prevent stochastic fade-out at the start of an epidemic have been previously discussed [35]). The high variation also suggests that control strategies based on *R*_0_ estimates generated from initial growth rates should be treated with caution and that estimations based on one particular setting might not be adequate to generalize and predict pathogen behaviour across all other spatial contexts [36].

We here concentrated solely on the basic reproduction number, which describes an arguably unusual and often artificial situation. However, it should be clear that the same arguments also hold for the effective reproduction number, *R*_*e*_, which is essentially *R*_0_ multiplied by the fraction of the population that is susceptible to a disease, as well as their time-dependent counterparts *R*(*t*) and *R*_*e*_(*t*). Furthermore, the serial and generation intervals, which can be understood as temporal analogues of the reproduction number, also rely on the vector to host transmission period and are usually assumed to be exponentially distributed [37, 38]. This implies that these intervals, and alternative *R*_0_ estimation methods that depend upon them [39–41], may equally be over-estimated.

Our work thus reiterates the importance of obtaining empirical vector mortality rates in the field. The original Ross-MacDonald model for the spread of *Plasmodium falciparum* and *P*. *vivax* malaria assumed constant vector mortality as laboratory and field studies seemed to suggest that death rates were age independent [42]. However, re-analysis of laboratory data showed that mosquito mortality is in fact age-dependent for several *Anopholes* species [43]. More recent studies also confirmed that mosquito mortality is dependent on age for *Anopheles* mosquitoes [27] and *Aedes aegypti* [24, 25]. What is clear is that more work needs to be done to fully elucidate realistic, i.e. field-relevant vector mortality rates, perhaps with more accurate spectroscopic methods [44], as well as their environmental drivers. That is, seasonal variations in temperature and rainfall have been shown to affect the birth and death rate of vectors [45–48], the vectorial competence [49] as well as the extrinsic incubation period [50–53]. It has also been emphasized that other spatio-temporal heterogeneities, such as community structures and host and vector movement, should be considered when assessing *R*_0_ [54, 55]. All this needs to be factored in if we are to develop better models to understand the epidemiological and ecological determinants of vector-borne diseases, guide outbreak prevention strategies or monitor ongoing intervention measures.

## Supporting information

S1 Text Model description and derivation of *R*_0_ estimates. (PDF)

S1 Fig Estimating *R*_0_ from empirical data. **(A)** The reproduction number can be estimated from epidemic outbreak data assuming an initially exponential growth rate, λ. **(B)** The dynamic equilibrium of susceptible individuals in a population can also be used to estimate *R*_0_.
(TIFF)

S2 FigSensitivity of *R*_0_ direct measurement on model parameters.The direct measurement of the mean number of secondary infections from a single introduction in the individual based model reliably estimates the theoretical calculation $R_{0}^{\text{IBM}}$. Longer vector life expectancies **(A)**, decreased age-dependence of vector mortality rates **(B)**, longer human infectious periods **(C)**, shorter extrinsic incubation periods **(D)**, and higher transmissibility **E–F**, increase $R_{0}^{\text{IBM}}$ and the mean number of secondary infections simulated in the individual based model. Each parameter was tested 2500 times, where the mean number of secondary infections was calculated from groups of 100 simulations. The dashed vertical lines represent the baseline values selected.(TIFF)

S3 Fig Sensitivity of stochastic fadeout on model parameters. The proportion of failed disease introductions in the individual based model depends upon a variety of factors. Longer vector life expectancies decrease the number of failed outbreaks **(A)**, whereas the age-dependence of vector mortality has little effect **(B)**. Longer human infectious periods **(C)**, shorter extrinsic incubation periods **(D)**, and higher transmissibility **(E–F)** naturally increase the overall likelihood of transmission from primary to secondary cases. Each parameter was simulated 2500 times, where the proportion of failed introductions was calculated from groups of 100 simulations. The dashed vertical lines represent the baseline values selected.
(TIFF)

S4 FigSensitivity of *R*_0_ estimates on model parameters.**(A)** Vector mortality shape parameter *c*_*V*_. Increasing the shape of the vector mortality distribution from constant to age-dependent survival rates shows that traditional theoretical approaches to *R*_0_ significantly overestimate the reproduction number $R_{0}^{\text{IBM}}$. Both estimates from the endemic equilibrium and post-correction initial growth rate continue to be robust over this range of shape parameters. **(B)** Vector mortality scale parameter *d*_*V*_. Both theoretical calculations scale linearly with the vector mortality scaling parameter, as this directly influences vector life expectancy and thus the vector-to-human transmission period (VHTP). Across all tested parameters, both estimates from the endemic equilibrium and post-correction initial growth rate continue to be reliable for *R*_0_ > 1. **(C)** Number of communities in the lattice |*C*|. The theoretical calculations of *R*_0_ presented do not explicitly contain any spatial dynamics. Increasing the number of communities (starting with a homogeneous mixing model) does not affect the robustness of *R*_0_ estimates. **(D)** External infection rate *ι*. The theoretical calculations of *R*_0_ presented do not explicitly contain the external infection rate. Increasing the external infection rate, does not influence the robustness of *R*_0_ estimates from the initial growth rate unless external infection rates are high enough to start driving the epidemiological dynamics. Furthermore, *R*_0_ estimates from the endemic equilibrium continue to be reliable until re-introduction of the disease into the system is too low for disease persistence. For each parameter value tested, 50 stochastic simulations were executed and *R*_0_ estimated for each simulation. The dashed vertical lines represents the baseline values selected.(TIFF)
