# Supplementary material for: Robustness of the reproductive number estimates in vector-borne disease systems
Source: PLoS Negl Trop Dis. 2018 Dec 17;12(12):e0006999. doi: 10.1371/journal.pntd.0006999 (PMC6312349; doi:10.1371/journal.pntd.0006999)
Supplement: S1 Text — (PDF) [file pntd.0006999.s001.pdf]

Robustness of the reproductive number estimates in vector-borne disease systems

Warren Tennant Mario Recker

Supplementary material

Individual Based Model

We employed a spatially-explicit IBM motivated by the one previously proposed by Lourenço and Recker (2013) [1].

Initially, a disease-free stochastic individual based model for the hosts and vectors of the virus was constructed with no spatial component, and time t discretized into days. Daily, individual humans and vectors were aged, and terminated if their age exceeded their life expectancy. Only the adult stage of a vector's life cycle was considered, since only adult vectors generally acquire and transmit the vector-borne disease. We assumed age-dependent mortalities for both the hosts and the vectors. For both the host and vector, this is governed a Weibull distribution.

$$\mu_i(t) = \frac{c_i}{d_i^{c_i}} t^{c_i - 1} e^{-\left(\frac{t}{d_i}\right)^{c_i}} \tag{1}$$

where c_i and d_i are the scale and shape parameters, with $i \in \{H, V\}$ denoting the parameters for host and vectors. The host and vector population were assumed to be constant, with M denoting the ratio if vectors to hosts, so a new individual immediately replaces an individual that has been the terminated.

Every host was defined to be either susceptible (S_H), exposed (E_H) (infected but not infectious), infectious (I_H), or have recovered from (R_H) the vector-borne disease. Similarly, we set vectors to be either susceptible (S_V), exposed (E_V) or infectious (I_V). Upon recovery of the vector-

borne disease, hosts are immune for the rest of their life. Susceptible hosts become exposed to the virus at a rate λ_H (force of infection), then become infectious after $1/\epsilon_H$ days, and finally recover after $1/\gamma$ days. Susceptible vectors become exposed to the virus at a rate λ_V, and develop infectiousness after $1/\epsilon_V$ days.

The force of infection on susceptible hosts, λ_H is dependent on the number of infected vectors in the system, and the force of infection on susceptible vectors, λ_V is dependent on the number of infected hosts in the system. All vectors bite hosts at a constant per day rate β. Thus the force of infection on susceptible hosts and on susceptible vectors are

$$\lambda_V(t) = p_V \beta \frac{I_H(t)}{N_H} \tag{2}$$

$$\lambda_H(t) = p_H \beta \frac{I_V(t)}{N_H} \tag{3}$$

where $p_{H,V}$ is the probability of successfully transmitting the disease given a biting event between an infected and susceptible individual, and $I_H(t)$ and $I_V(t)$ are the number of hosts and vectors infected at time t respectively. Additionally, individuals were infected at an external infection rate ι to account for introduction of the disease from outside the system.

The host and vector populations were divided into a set of communities organized into a non-wrapping lattice. The distribution of humans and mosquitoes was set to be maximally uniform across all communities. We assumed that individuals mix homogeneously within each community. Let C be the set of all communities in the meta-population and define $\vec{c} = [r, c] \in C$ where $[r, c]$ is the community's row and column number in the lattice. In a model with no inter-community interaction, communities are isolated from one another, so the force of infection terms of each community $\vec{c} \in C$ are

$$\lambda_V(t, \vec{c}) = p_V \beta \frac{I_H(t, \vec{c})}{N_H(\vec{c})} \tag{4}$$

$$\lambda_H(t, \vec{c}) = p_H \beta \frac{I_V(t, \vec{c})}{N_H(\vec{c})} \tag{5}$$

where $I_H(t, \vec{c})$ and $I_V(t, \vec{c})$ are the number of infected hosts and vectors respectively in community $\vec{c}$ at time t, and $N_H(\vec{c})$ is the number of humans in community $\vec{c}$.

Infection between communities was considered by dispersing the total number of transmission

events in a community to neighbouring communities, thereby modelling the biting of vectors in surrounding sub-populations. The total number of transmissions in community $\vec{c} \in C$ at time t is the force of infection multiplied by the population size. Inter-community transmissions were included by setting the total number of transmissions for each community to be a weighted sum of the total number of transmissions for each sub-population in a model where all communities are isolated, with weights defined by some disease dispersal kernel $\Phi(\cdot)$.

$$N_V(\vec{c})\,\Lambda_V(t,\vec{c}) = \sum_{\vec{\zeta}\in C} \Phi_{\vec{\zeta}}(\vec{c})\,\lambda_V\left(t,\vec{\zeta}\right) N_V\left(\vec{\zeta}\right) \tag{6}$$

$$N_H(\vec{c})\,\Lambda_H(t,\vec{c}) = \sum_{\vec{\zeta}\in C} \Phi_{\vec{\zeta}}(\vec{c})\,\lambda_H\left(t,\vec{\zeta}\right) N_H\left(\vec{\zeta}\right) \tag{7}$$

where $\Phi_{\vec{\zeta}}(\vec{c})$ are the normalized weights, $N_V(t,\vec{c})$ and $N_H(t,\vec{c})$ are the total number of vectors and hosts respectively in community $\vec{c}$ at time t, and $\Lambda_V(t,\vec{c})$ and $\Lambda_H(t,\vec{c})$ are the force of infection terms on vectors and hosts respectively in community $\vec{c}$ at time t with inter-community transmissions.

The local disease dispersal kernel is $\Phi_{\vec{\zeta}}$ is normalized and dependent on the distance between the source community $\vec{\zeta}$ and each destination community $\vec{c} \in C$ involved in the dispersal of each transmission event. It was assumed that the distance dependence follows a normal distribution centred at mean zero with variance σ^2. Therefore, $\phi_{\vec{\zeta}}(\vec{c})$ can be computed as the volume of the two dimensional normal distribution centred around $\vec{\zeta}$ with covariance matrix $\mathbf{\Sigma}$ evaluated at the community $\vec{c}$.

A model for human movement was incorporated into the model by allowing human individuals to temporarily visit any sub-population in the lattice with probability ω. Combining this with the local disease dispersal kernel, the total number of transmission events for vectors and hosts are computed as

$$N_V(t,\vec{c})\,\Lambda_V(t,\vec{c}) = \sum_{\vec{\zeta}\in C} \left[(1-\omega)\,\Phi_{\vec{c}}\left(\vec{\zeta}\right) + \frac{\omega}{|C|}\right] \lambda_V\left(t,\vec{\zeta}\right) N_V\left(t,\vec{\zeta}\right) \tag{8}$$

$$N_H(\vec{c})\,\Lambda_H(t,\vec{c}) = \sum_{\vec{\zeta}\in C} \left[(1-\omega)\,\Phi_{\vec{c}}\left(\vec{\zeta}\right) + \frac{\omega}{|C|}\right] \lambda_H\left(t,\vec{\zeta}\right) N_H\left(\vec{\zeta}\right) \tag{9}$$

where $|C|$ is the total number of communities in the lattice.

The mathematical method was implemented computationally in C++ with CUDA GPU acceleration in order to reduced simulation run-time such that extensive sensitivity analyses could be performed.

Computational implementation

The mathematical method was implemented computationally in C++ with CUDA GPU acceleration in order to reduced simulation run-time such that extensive sensitivity analyses could be performed.

In the model, a human individual is defined by their age, a random probability determining an individuals life expectancy, the community in which they reside, and the immunological history of the individual, what infected class they are in (susceptible, exposed, infected). An infected or exposed human individual will also the age at which they become infectious, and the age at which they recover. Similarly, an individual mosquito is defined by their age, the random probability, and home community. Infected mosquito also have information on the age at which they become infectious. Note that by the compartmental model in the previous subsection, there is no need for recovery times or immunological history since a mosquito is infected for life.

Firstly, a disease free human and mosquito population are initialized. An individuals age is initialized from the survival function, the probability that an individual will survive beyond a given age. For an age-dependent mortality rate $\mu(t)$, the survival function $S(t)$ is given by

$$s(t) = 1 - \int_0^t \mu(\tau)\, d\tau \tag{10}$$

The life expectancy probability is assigned on a uniform distribution of $[s(t), 1]$. Individuals are assigned communities uniformly. A small proportion ($\approx 0.01\%$) of individuals are then infected with infected humans being assigned an age at which they recover.

Daily, individuals are subject to a demographic update and viral transmissions, as well as some data collation, up until the requested simulation time (not runtime). For the demographic update, an cumulative probability function of the mortality risk at the individuals age is compared with their life expectancy probability. If the cumulative probability exceeds the life expectancy probability, the individual is terminated. A new human always replaces a deceased human im-

mediately, whereas a dead mosquito is replaced conditional on the expected mosquito population size on a given simulation day (rainfall seasonality). In the demographic update, individuals are also checked as to if they should become infectious or if humans should recover from the disease. The demographic update code fragment also contains counters in order to keep track of the total number of infected individuals per community.

For viral transmissions, for host and vector, the expected number of transmission events from each sub-population are computed (λN) and divided between into long-distance and local transmissions. Each long-distance transmission event is assigned community which is uniformly sampled from the lattice. Every local transmission is assigned a community which is sampled from the local disease dispersal kernel. After all transmission events from each sub-population have been passed through the inter-community interaction function, each community now has a new total number of transmissions pass to its individuals (ΛN). For every transmission event in a community, an individual is selected at random. If the individual is not already infected, and does not have immunity, the individual is infected. A newly infected mosquito is assigned an extrinsic incubation period, at the end of which the mosquito becomes infectious for the duration of its life. Similarly, a human is assigned an intrinsic incubation period and a recovery time, and is now set to be immune.

Finally, at every time step, the counters keeping track of the total number of infected individuals per community are summed. This data is output at the very end of the program.

GPU acceleration

The majority of the code is parallelized on the GPU. The parts that are not parallelized include requesting user input, checking whether the simulation needs to continue, and outputting data to file. The demographic update part of the code is fully parallelized. This means that every scheduled thread on the GPU is associated with an individual. Computing the expected number of viral transmissions per community per strain (before and after local and long-distance dispersal) is parallelized across communities. However, infection of individuals happens also at the community level. The summation of counters across all sub-populations is parallelized over all communities.

Optimization

Since all individuals require updating at every time-step of the simulation, the demographic update is the main target for optimization. Defining an individual as an object and arranging many individuals into a vector results in strided memory access when accessing one piece of information about each individual, such as age or location. A noted optimization is to break down the vector of individuals into vectors of component parts. Memory access is now coalesced. There are only two kernel calls for the demographic update; one for mosquitoes, and one for humans. Each kernel is not sub-divided into smaller functions in order to reduce the number of memory stores and loads. It is expected that the mosquito demographic update is slower than the human update since the much higher per day death rate in mosquitoes leads to a large increase in warp divergence. Other simpler optimization strategies suggested in the CUDA manual, such as using fast math libraries, were followed throughout the parallelized parts of code.

ODE-based reproductive number

We applied the next generation approach [2] to a typical SEIR-SEI host-vector system of ordinary differential equations in order to get the formula for the basic reproductive number R_0^{ODE}.

Firstly, by assuming the entire host and vector population is susceptible to the pathogen, the ODEs (see Methods in the main text) can be reduced to the following subsystem.

$$\frac{dE_H}{dt} = p_H \beta I_V - \epsilon_H E_H - \mu_H E_H \tag{11}$$

$$\frac{dI_H}{dt} = \epsilon_H E_H - \gamma I_H - \mu_H I_H \tag{12}$$

$$\frac{dE_V}{dt} = M p_V \beta I_H - \epsilon_V E_V - \mu_V E_V \tag{13}$$

$$\frac{dI_V}{dt} = \epsilon_V E_V - \mu_V I_V \tag{14}$$

where M is the number of vectors per host (N_V/N_H). Then, by setting $\boldsymbol{x} = [E_H, I_H, E_V, I_V]^T$, the subsystem can be written as

$$\dot{\boldsymbol{x}} = (\boldsymbol{T} + \boldsymbol{\Sigma})\, \boldsymbol{x} \tag{15}$$

$$\boldsymbol{T} = \begin{bmatrix} 0 & 0 & 0 & p_H\beta \\ 0 & 0 & 0 & 0 \\ 0 & Mp_V\beta & 0 & 0 \\ 0 & 0 & 0 & 0 \end{bmatrix} \qquad \boldsymbol{\Sigma} = \begin{bmatrix} -(\epsilon_H+\mu_H) & 0 & 0 & 0 \\ \epsilon_H & -(\gamma+\mu_H) & 0 & 0 \\ 0 & 0 & -(\epsilon_V+\mu_V) & 0 \\ 0 & 0 & \epsilon_V & -\mu_V \end{bmatrix} \tag{16}$$

where $\boldsymbol{T}$ represents the transmission of the pathogen between hosts and vectors, and $\boldsymbol{\Sigma}$ corresponds to the transitions of individuals between exposed and infected classes. The next generation matrix K_L can then be calculated.

$$K_L := -T\Sigma^{-1} = \begin{bmatrix} 0 & 0 & \frac{p_H\beta\epsilon_V}{(\epsilon_V+\mu_V)\mu_V} & \frac{p_H\beta}{\mu_V} \\ 0 & 0 & 0 & 0 \\ \frac{Mp_V\beta\epsilon_H}{(\epsilon_H+\mu_H)(\gamma+\mu_H)} & \frac{Mp_V\beta}{\gamma+\mu_H} & 0 & 0 \\ 0 & 0 & 0 & 0 \end{bmatrix} \tag{17}$$

Diekmann (2010) showed that dominant eigenvalue of the next generation matrix is the reproductive number of the original system of ordinary differential equations. This eigenvalue is the square root of the reproduction number for the full transmission cycle of a vector-borne disease, which is what we are interested in. Therefore

$$R_0 = \frac{Mp_Hp_V\beta^2}{(\gamma+\mu_H)\,\mu_V} \frac{\epsilon_H}{\epsilon_H+\mu_H} \frac{\epsilon_V}{\epsilon_V+\mu_V} \tag{18}$$

However, for most vector-borne diseases the human recovery rate $1/\gamma$, and intrinsic incubation period $1/\epsilon_H$, are much shorter than mean human life expectancy $1/\mu_H$ in the ODE system, an approximation can be made by removing the death rate of humans μ_H from the formula

$$R_0^{\text{ODE}} = \frac{Mp_Hp_V\beta^2}{\gamma} \frac{1}{\mu_V} \frac{\epsilon_V}{\epsilon_V+\mu_V} \tag{19}$$

IBM-based reproductive number

The basic reproductive number, R_0^{IBM}, for the individual based model was computed from first principles using the transmission cycle of the pathogen as described by the individual-based model itself. Starting with an infected human in an entirely susceptible population, the human

will infect $Mp_V\beta$ vectors per day on average and will be infected for $1/\gamma$ days. Therefore, a single infected human is expected of infect $Mp_V\beta/\gamma$ vectors.

The proportion of infected vectors that survive the extrinsic incubation period of the disease is denoted by $\rho_{E_V \to I_V}$ and was calculated from the vector mortality risk μ_V. First, we calculate the survival function $s(\tau)$. The survival function is a monotonically decreasing function which defines the probability that a vector will survive beyond τ days in age.

$$s(\tau) = 1 - \int_0^\tau \mu_V(t)\, dt \tag{20}$$

The probability that an individual of age τ will survive a mean incubation period of $1/\epsilon_V$ is denoted by $\rho_{E_V \to I_V}(\tau)$.

$$\rho_{E_V \to I_V}(\tau) = \frac{s\left(\tau + \frac{1}{\epsilon_V}\right)}{s(\tau)} \tag{21}$$

In order to get the mean probability that an infected vector becomes infectious, we weight $\rho_{E_V \to I_V}(\tau)$ by the proportion of the vector population at age τ, derived from the normalization of the survival function.

$$\rho_{E_V \to I_V} = \sum_{\tau=0}^{\infty} \left(\rho_{E_V \to I_V}(\tau) \frac{s(\tau)}{\sum_{\hat{\tau}=0}^{\infty} s(\hat{\tau})} \right) \tag{22}$$

$$= \sum_{\tau=0}^{\infty} \left(\frac{s\left(\tau + \frac{1}{\epsilon_V}\right)}{s(\tau)} \frac{s(\tau)}{\sum_{\hat{\tau}=0}^{\infty} s(\hat{\tau})} \right) \tag{23}$$

$$= \frac{\sum_{\tau=0}^{\infty} s\left(\tau + \frac{1}{\epsilon_V}\right)}{\sum_{\hat{\tau}=0}^{\infty} s(\hat{\tau})} \tag{24}$$

A single infected vector will infect $p_H\beta$ humans per day on average. As infectious vectors can infect for the rest of their life, the infectious period is defined as difference between the mean life expectancy of an infectious vector, $1/\mu_{I_V}$, and the mean age at which a vector becomes infectious, $1/\alpha_{I_V}$. Therefore, the infectious period is dependent upon the vector mortality rate.

As the vector population is assumed to be completely susceptible in the definition of R_0, the mean age of an infectious vector is calculated by taking the weighted average of all possible ages of an infectious vector, $\tau + 1/\epsilon_V$, with the proportion of infectious vectors at that given age,

obtained by the normalization of the survival function perturbed by the incubation period.

$$\frac{1}{\alpha_{I_V}} = \sum_{\tau=0}^{\infty} \left(\tau + \frac{1}{\epsilon_V}\right) s\left(\tau + \frac{1}{\epsilon_V}\right) \frac{1}{\sum_{\hat{\tau}=0}^{\infty} s\left(\hat{\tau} + \frac{1}{\epsilon_V}\right)} \tag{25}$$

The mean life expectancy of an infectious vector, $1/\mu_{I_V}$, is not the same as the mean life expectancy of a vector, because a vector that became infectious at time $\tau + 1/\epsilon_V$ must have survived up until that age, thereby altering its remaining mortality risk distribution. Therefore, the mean life expectancy of an infectious vector that became infected at age τ days, $f(\tau)$, must survive at least $\tau + 1/\epsilon_V$ plus the remaining expected survival time.

$$f(\tau) = \tau + \frac{1}{\epsilon_V} + \frac{1}{s\left(\tau + \frac{1}{\epsilon_V}\right)} \sum_{T=0}^{\infty} s\left(\tau + \frac{1}{\epsilon_V} + T\right) \tag{26}$$

where $s\left(\tau + \frac{1}{\epsilon_V}\right)^{-1}$ rescales the remaining survival distribution to be the probability of survival given the vector survived until $\tau + 1/\epsilon_V$ days. As the individual based model discretizes time into days, the remaining survival period can be calculated by taking the sum over all possible (infectious) ages of the remaining survival function.

Again, by taking the weighted average of all possible ages of an infectious vector, $\tau + 1/\epsilon_V$, with the mean life expectancy of a vector that became infectious at age $\tau + 1/\epsilon_V$ days, we obtain an the mean life expectancy of an infectious vector.

$$\frac{1}{\mu_{I_V}} = \sum_{\tau=0}^{\infty} f(\tau) \frac{s\left(\tau + \frac{1}{\epsilon_V}\right)}{\sum_{\hat{\tau}=0}^{\infty} s\left(\hat{\tau} + \frac{1}{\epsilon_V}\right)} \tag{27}$$

Combining all these terms, the basic reproduction number of the individual based model can be derived as

$$R_0^{\mathrm{IBM}} = \frac{M p_H p_V \beta^2}{\gamma} \left(\frac{1}{\mu_{I_V}} - \frac{1}{\alpha_{I_V}}\right) \rho_{E_V \to I_V} \tag{28}$$

Basic reproduction number from initial growth rate

The basic reproduction number can be estimated from the time-series of an outbreak of a vector-borne disease in an entirely susceptible population of hosts and vectors.

By assuming that the proportion of infected hosts ($i = H$) and vectors ($i = V$) survive the intrinsic and extrinsic incubation period is $\epsilon_i / (\epsilon_i + \mu_i)$, and that the entire host and vector populations are susceptible to the vector-borne disease the infectious stage of the SEIR-SEI ODE system can be rewritten into the following delay differential equation

$$\dot{I_H} = \frac{\epsilon_H}{\epsilon_H + \mu_H} p_H \beta I_V (t - \tau_V) - (\gamma + \mu_H) I_H \tag{29}$$

$$\dot{I_V} = \frac{\epsilon_V}{\epsilon_V + \mu_V} M p_V \beta I_H (t - \tau_H) - \mu_V I_V \tag{30}$$

where $\tau_H = 1/\epsilon_H$ and $\tau_V = 1/\epsilon_V$, are the intrinsic and extrinsic incubation periods respectively.

At the start of an epidemic, the growth rate of infected hosts and vectors is assumed to be a constant λ, so the total number of infected hosts and vectors is defined as

$$I_H (t) = c_H \exp (\lambda t) \tag{31}$$

$$I_V (t) = c_V \exp (\lambda t) \tag{32}$$

Substituting these into equations 29 and 30 and re-arranging yields

$$1 = \frac{\epsilon_H}{\epsilon_H + \mu_H} \frac{\epsilon_V}{\epsilon_V + \mu_V} \frac{M p^2 \beta^2}{(\lambda + \gamma + \mu_H)(\lambda + \mu_V)} \exp (-\lambda (\tau_V + \tau_H)) \tag{33}$$

And then substituting the equation for R_0 derived from the ODE system using the next-generation method yields

$$R_0^{(\lambda)} = \left(1 + \frac{\lambda}{\gamma + \mu_H}\right) \left(1 + \frac{\lambda}{\mu_V}\right) \exp (\lambda (\tau_V + \tau_H)) \tag{34}$$

Equilibrium estimation methods are more reliable than using the initial growth rate

The asymptotically stable steady state of susceptible individuals in an ODE-based SEIR system for a directly transmitted disease is a good estimator of the basic reproductive number and is given as

$$R_0^* = \frac{1}{S^*} \tag{35}$$

where S^* is the mean proportion of susceptible individuals at equilibrium. The directly transmitted disease R_0 estimate was then used as an approximation for the basic reproductive number of an endemic vector-borne disease. For the individual based model (IBM) this required the proportion of susceptible individuals to reach some dynamic equilibrium, as the inherent stochasticity inhibits the system from reaching an asymptotically stable equilibrium (Figure A in S1 Figure).

Under the assumption of constant vector mortality rate, estimating the reproduction number from the dynamic equilibrium of susceptible individuals is much more reliable than from the initial growth rate of an epidemic in an entirely susceptible population (Figure B in S1 Figure). Although the estimate is more robust, gathering seroprevalence data in the field offers substantially greater challenges than collecting the total number of cases over time at the start of an outbreak.

References

[1] Lourenço J, Recker M. Natural, Persistent Oscillations in a Spatial Multi-Strain Disease System with Application to Dengue. PLoS Computational Biology. 2013;9(10). doi:10.1371/journal.pcbi.1003308.

[2] Diekmann O, Heesterbeek JAP, Roberts MG. The construction of next-generation matrices for compartmental epidemic models. Journal of The Royal Society Interface. 2010;7(47):873–885. doi:10.1098/rsif.2009.0386.
