# Supplementary figures and images for: Robustness of the reproductive number estimates in vector-borne disease systems
Source: PLoS Negl Trop Dis. 2018 Dec 17;12(12):e0006999. doi: 10.1371/journal.pntd.0006999 (PMC6312349; doi:10.1371/journal.pntd.0006999)

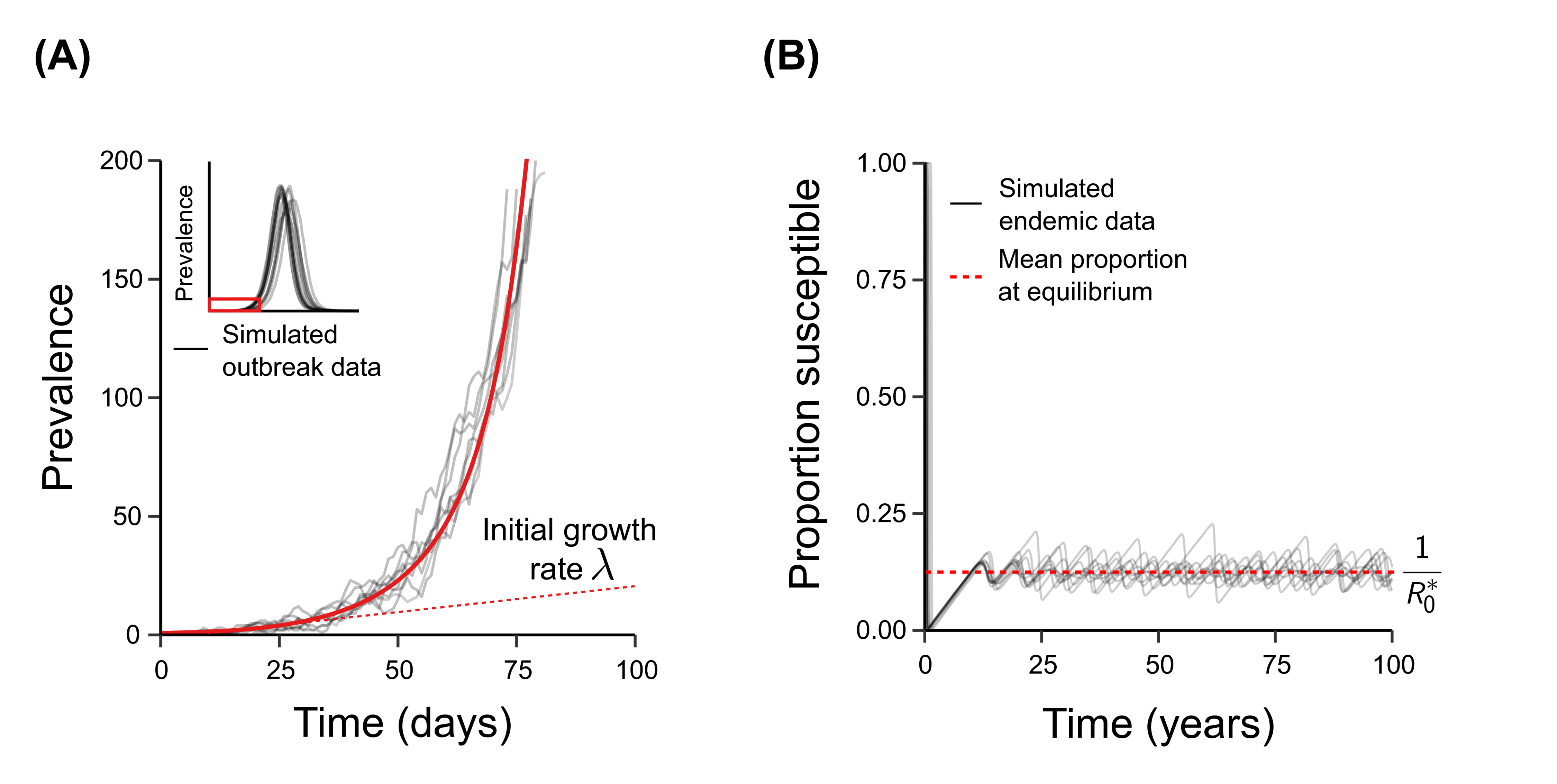

Supplement: S1 Fig — (A) The reproduction number can be estimated from epidemic outbreak data assuming an initially exponential growth rate, λ. (B) The dynamic equilibrium of susceptible individuals in a population can also be used to estimate R0. (TIFF) [file pntd.0006999.s002.tiff]

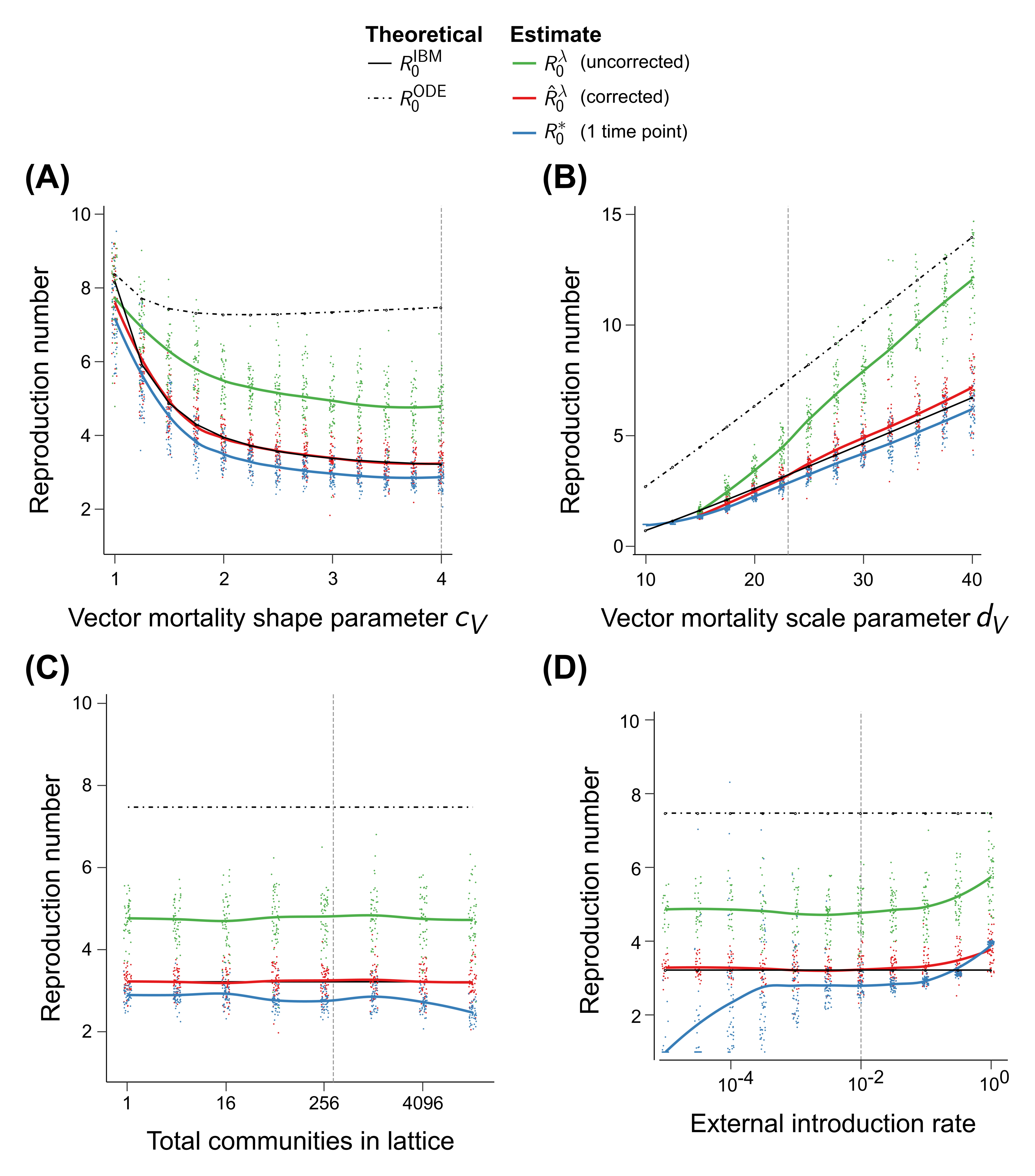

Supplement: S4 Fig — (A) Vector mortality shape parameter cV. Increasing the shape of the vector mortality distribution from constant to age-dependent survival rates shows that traditional theoretical approaches to R0 significantly overestimate the reproduction number R0IBM. Both estimates from the endemic equilibrium and post-correction initial growth rate continue to be robust over this range of shape parameters. (B) Vector mortality scale parameter dV. Both theoretical calculations scale linearly with the vector mortality scaling parameter, as this directly influences vector life expectancy and thus the vector-to-human transmission period (VHTP). Across all tested parameters, both estimates from the endemic equilibrium and post-correction initial growth rate continue to be reliable for R0 > 1. (C) Number of communities in the lattice |C|. The theoretical calculations of R0 presented do not explicitly contain any spatial dynamics. Increasing the number of communities (starting with a homogeneous mixing model) does not affect the robustness of R0 estimates. (D) External infection rate ι. The theoretical calculations of R0 presented do not explicitly contain the external infection rate. Increasing the external infection rate, does not influence the robustness of R0 estimates from the initial growth rate unless external infection rates are high enough to start driving the epidemiological dynamics. Furthermore, R0 estimates from the endemic equilibrium continue to be reliable until re-introduction of the disease into the system is too low for disease persistence. For each parameter value tested, 50 stochastic simulations were executed and R0 estimated for each simulation. The dashed vertical lines represents the baseline values selected. (TIFF) [file pntd.0006999.s005.tiff]
